# Hexagonal Stimulation Digital Controller Design and Verification for Wireless Subretinal Implant Device

**DOI:** 10.3390/s22082899

**Published:** 2022-04-10

**Authors:** Wajahat Abbasi, Hojong Choi, Jungsuk Kim

**Affiliations:** 1 Department of Health Science and Technology, Gachon Advanced Institute for Health Sciences and echnology, Incheon 21999, Korea; wajahat@bme.gachon.ac.kr; 2 Department of Electronic Engineering, Gachon University, 1342 Seongnam-daero, Seongnam 13120, Korea; 3 Department of Biomedical Engineering, Gachon University, 191 Hambakmoe-ro, Incheon 21936, Korea

**Keywords:** field-programmable gated arrays, digital controller, wireless telemetry system, hexagonal stimulation

## Abstract

Significant progress has been made in the field of micro/nano-retinal implant technologies. However, the high pixel range, power leakage, reliability, and lifespan of retinal implants are still questionable. Active implantable devices are safe, cost-effective, and reliable. Although a device that can meet basic safety requirements set by the Food and Drug Administration and the European Union is reliable for long-term use and provides control on current and voltage parameters, it will be expensive and cannot be commercially successful. This study proposes an economical, fully controllable, and configurable wireless communication system based on field-programmable gated arrays (FPGAs) that were designed with the ability to cope with the issues that arise in retinal implantation. This system incorporates hexagonal biphasic stimulation pulses generated by a digital controller that can be fully controlled using an external transmitter. The integration of two separate domain analog systems and a digital controller based on FPGAs is proposed in this study. The system was also implemented on a microchip and verified using in vitro results.

## 1. Introduction

Vision plays an important role in the transfer of external information from the surroundings to the brain, constituting almost 90% of all external information. Photoreceptors in the retinal layer of the eye are responsible for converting photons into neural signals that are transmitted to the brain for the generation of vision. Age macular degeneration (AMD) and retinitis pigmentosa (RP) are diseases that cause photoreceptor dysfunction and lead to loss of vision, night blindness, and eventually blindness.

Functional electric stimulation (FES) has been widely used in various studies to restore human vision and has gained a reputation for restoring vision loss caused by RP and AMD. The focus of FES is to deliver an electric charge to depolarize the neural membrane. A critical aspect of FES while generating an action potential at the neural membrane is controlling the electric charge, which includes specific parameters, such as the current value and shape of the current pulses.

Advancements in retinal prosthesis have resulted in various techniques based on the anatomical position of the implanted electrode array inside the retina, which can be classified as epi-retinal [1,2,3], sub-retinal [4,5,6,7], and suprachoroidal [6,8] devices. Subretinal implants can provide high pixel densities of up to 1600 pixels [9]. Several shapes of stimulation pulses [6,10,11,12,13,14] have been proposed to increase the stimulation efficiency, reduce energy requirement, and prevent current leakage [14,15,16,17].

The rectangular biphasic pulse [10,11,12,13,14] has evolved to be a solution to the most critical parameters, that is, stimulation efficiency and prevention of amplitude mismatch to avoid current leakage. In addition, each visually impaired patient has a different threshold for activating retinal neurons; therefore, it is critical to control the size, shape, and amplitude of current pulses using an embedded digital controller or stimulus generator. Cross-talk is generated between the electrodes in the stimulator by an electric field generated during stimulation. Retinal tissues are damaged due to this cross-talk, and unwanted neighboring electrodes could be stimulated [18,19].

The control of the currents in retinal tissues has remained a challenge [20,21]. Many systems use a single reference electrode, which creates a long distance between the active and reference electrodes. This distance elicits other electrodes in the vicinity, that can excite unwanted tissues. This excitation causes a current loss, and therefore, systems can cause damage inside the eye.

Many approaches have been proposed to address this issue, each with its own set of drawbacks. In various approaches, analog current control methods have been used, which ultimately lead to current leakage and power dissipation and do not provide complete control over current, resulting in tissue damage. On the contrary, the methods used to elicit electrodes cause cross-talk among electrodes, which was proven through simulation results using COMSOL in this study.

To overcome these drawbacks, our system proposes hexagonal stimulation [11] to counter the loss of current [11] and reduce cross-talk between the electrodes. A 64-pixel electrode array was stimulated in this approach using the hexagonal stimulation of the electrodes.

The aim of this study is to effectively introduce a digital controller that controls every operation of hexagonal stimulations. It is responsible for the generation of specific data packets [3] based on the light along with a 2 MHz clock that is generated within the transmitter. These data and the clock are responsible for the complete control of the biphasic pulses on the receiver side that elicit the electrodes within the stimulator with relief for current control and current leakage prevention.

Our wireless [22,23,24] subretinal system comprises three parts as follows:

1. Design of an external power and data transmitter that supplies power to the implanted retinal system and light-dependent data for controlling the stimulation pulses;
2. The implanted power and data receiver recovers data and power from the external transmitter, generates stimulation pulses of various shapes based on light-dependent data, and delivers it to the stimulator;
3. An electrode stimulator activates neurons of the retina.

**Figure 1:**
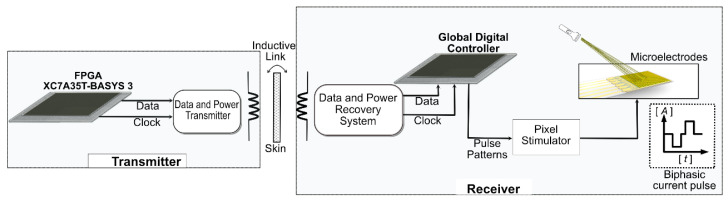
Block diagram of the system. XC7A35T version FPGA is used to generate data and clock signals on the transmitter side and is transferred through an inductive link. The receiver comprises data and power recovery systems, a global digital controller, and a pixel simulator.

A block diagram of the system is shown in Figure 1.

## 2. Materials and Methods

There has long been a need for retinal systems that cover all the fundamental aspects of vision enhancement while considering electrical and biological factors. The aim of this system is to bring it closer to real-world applications. This designed architecture comprised four main parts: a transmitter that generated clock and data outputs dependent on external light using a field-programmable gated array (FPGA) BASYS 3. The generated data were based on a specific protocol and were transmitted in the form of data packets, which ensured the correct encoding of data on the receiver side. A wireless data telemetry system was designed that transmitted a 2 MHz clock signal, 32-bit data packets at a frequency of 10 kHz, and sufficient power, which was later recovered on the receiver side. This prevented the receiver from relying on batteries or internal power; instead, it worked by using the power received through the transmitter. A global digital controller that received clock and data signals was responsible for generating specific pulse patterns to generate the biphasic pulse and was driven by external power received through wireless power transfer. A stimulator pixel comprised a photosensor, a current amplifier, and a pulse shaper that generated biphasic pulses in response to specific pulse patterns generated within the digital controller.

### 2.1. Hexagonal Stimulation

**Figure 2:**
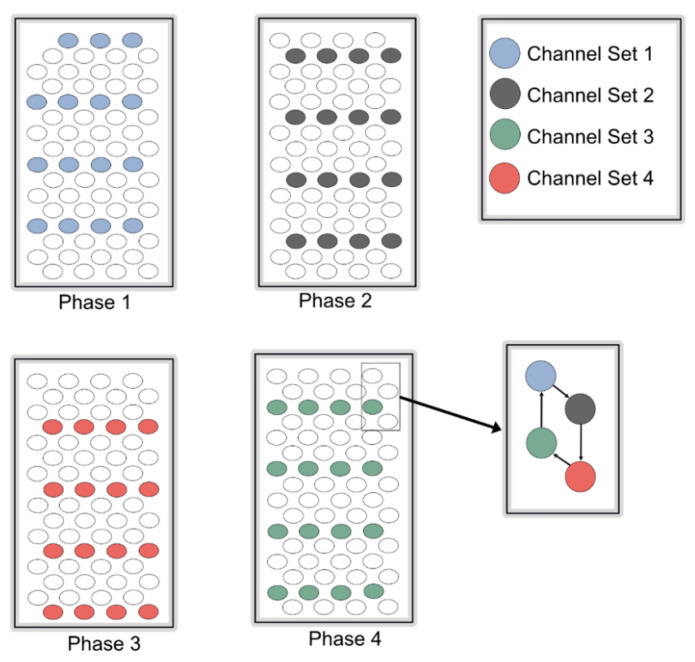
Pattern of electrode stimulation. Each phase shows a specific interval of stimulations of a single channel set, while hexagonal pattern of stimulation is shown in a small box.

Controlling current loss is one of the major factors affecting eye tissue damage. This system consists of a hexagonal stimulator, which is entirely controlled by an on-chip digital controller, as explained in the following section. Figure 2 shows the structure of the hexagonal stimulator. A total of 64 electrode pixels were divided into 4 channel sets, each comprising 16 pixels. In this system, 4 phases excited 64 pixels, whereas 16 pixels were excited in 1 phase. When a single channel set was turned on for its respective time, 16 pixels were excited, and the remaining electrode pixels acted as a reference (ground). Therefore, this technique of eliciting electrode pixels reduces the artifacts caused by current leakage or cross-talk.

Hexagonal stimulation was performed using biphasic pulses, which tend to be more beneficial than other pulse shapes. A global digital controller was used to generate biphasic pulses.

### 2.2. Global Digital Controller

A global digital controller, which is part of a complete system, comprises two main systems: transmitter and receiver. The individual system architecture of the transmitter and receiver, which acts as a global digital controller, is described in what follows.

#### 2.2.1. Transmitter

This system uses near-field communication for the transmission of data and clock outputs through a wireless data telemetry system; therefore, a single data transfer protocol was used for all the devices. These data were received and processed on the receiver side to generate various pulse patterns, which are explained in detail in this section.

**Figure 3:**
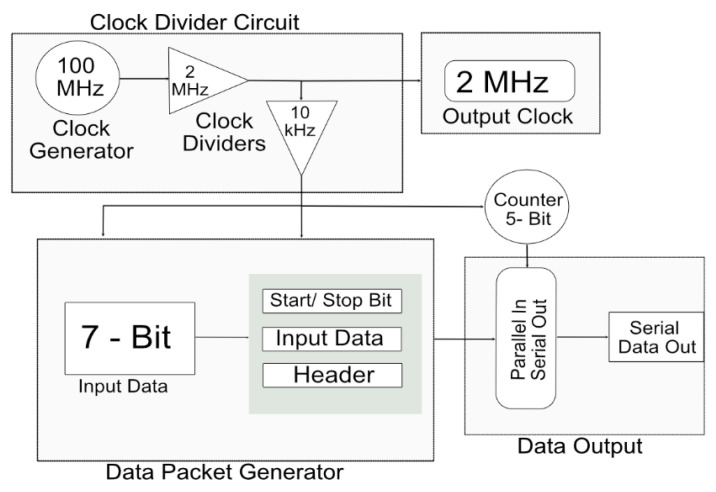
Detailed architecture of transmitter for wireless data telemetry system.

An important consideration when designing a transmitter is that data should be light-dependent, and every transmitted data packet should represent a specific intensity of light. For the current system, a prototype FPGA (BASYS 3) was used to generate data. Subsequently, the proposed system depends on various intensities of light. The data were transmitted in the binary form 0 s and 1 s. A clock was generated on the transmitter FPGA at a rate of 100 MHz. The clock could be divided based on the requirements of the global digital controller on the stimulator side. The various speeds of the clock made the system more reliable because the total stimulation period could be varied on the transmitter side. A single clock with a frequency of 2 MHz was used for the current system. Figure 3 shows the proposed complete transmitter system.

The transmitter generated an internal clock at a speed of 100 MHz. Clock dividers were used to attain the desired frequency of 2 MHz from the 100 MHz built-in clock of the FPGA. This 2 MHz clock was output from the transmitter to the receiver through a wireless transmitter system for the generation of pulse profiles and synchronization processes.

Seven-bit external data were input to the transmitter FPGA. The data were input in the form of 0 s and 1 s, which gave a total of 128 combinations. Each combination was responsible for the shape and amplitude of the biphasic pulse. The input data were combined with the start bit, 24-bit header, and stop bit. The start bit indicated the start of the new data packet. Each data pattern had a specific header that provided the address of the data and verifies the data packet. The stop bit acted as parity and indicated the end of the data packet. The 32-bit parallel data were input into a multiplexer that converted it into serial data. A five-bit counter acted as a selection line for the multiplexer and each bit of the data packet was transferred to each pattern of the selection line.

#### 2.2.2. Data Packet Structure

**Figure 4:**
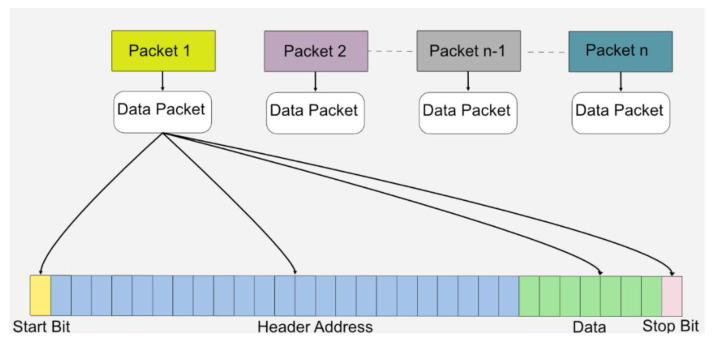
The packet format protocol used in this system for wireless data transmission.

Data on the transmitter side were generated based on the light transmitted through the FGPA. For data transmission, a specific data packet was generated, which included a start bit indicating the start of a new packet, followed by a specific, fixed 23-bit address of the data. This indicated the type of data transmitted and verified the correct data packet. Once the header was verified, seven-bit variable data were processed, and the stop bit, which also acted as parity, indicated the end of the data packet. Figure 4 shows the data packet structure.

The data packet comprised 32 bits. In addition, 25 bits were used for the transmission and verification of data packets by following a simple verification protocol. Seven-bit binary data were generated based on the light intensity, with an initial value starting from decimal value “0” and maximum value extending to maximum decimal value “127”. Each data value changed the shape and amplitude of the pulse patterns as the light intensity changed. In addition, it also provided manual control for the subject based on the intensity of light in case the subject wanted a specific change in the amplitude or shape of pulses that would elicit electrodes inside the human eye. The data packet structure is shown in Figure 4.

#### 2.2.3. Wireless Data and Power Telemetry

Wireless communication uses near-field communication. Wireless telemetry systems use an inductive coil, which is a popular method for transferring wireless data and power. In the current system, the external device comprised a class-E power amplifier, amplitude-shift-keying (ASK) modulator circuit, and current sense circuit for back-telemetry data recovery. The implanted device had a rectifier, regulator, overvoltage protection circuit, demodulator, and reverse telemetry controller. The class-E amplifier was driven by a 2 MHz carrier signal, and data were modulated on the carrier signal, which was then transmitted through an inductive link. The received signal in the implanted device was rectified and fed into the regulator to generate dual-polarity supply voltages. The global digital controller started operating when clock and data signals were received from the demodulator. The status of the implanted stimulators was observed from the external side by generating back-telemetry data. Load-shift keying (LSK) through an inductive link technique was used for back-telemetry, which was fully controlled by a reverse telemetry controller. The overvoltage protection circuit in the implanted device was activated when the rectified signal exceeded the allowable voltage limit. This circuit shifted the reactance of the implanted coil and capacitor away from its resonance; as a result, the received signal was attenuated at a safe voltage level.

#### 2.2.4. Digital Controller

The data and clock outputs were received by the global digital controller after they were processed through a wireless data telemetry system and recovered using a demodulator. The operation of the wireless data telemetry system was explained in the previous section. The global digital controller used the data and clock signals received from the transmitter to generate specific pulse patterns responsible for the generation of a biphasic pulse. Three testing platforms for the global digital controller were used for testing the complete wireless telemetry system. ModelSim (Mentor Graphics, Wilsonville, OR, USA) was used to perform the simulations. The results were verified using hypothetical simulations. Hardware was initially implemented on the FPGAs to verify the results. The FPGAs were used to transmit and receive data, and the results were posted on an oscilloscope. A microchip designed for both a 64-pixel stimulator and its global digital controller was fabricated.

**Figure 5:**
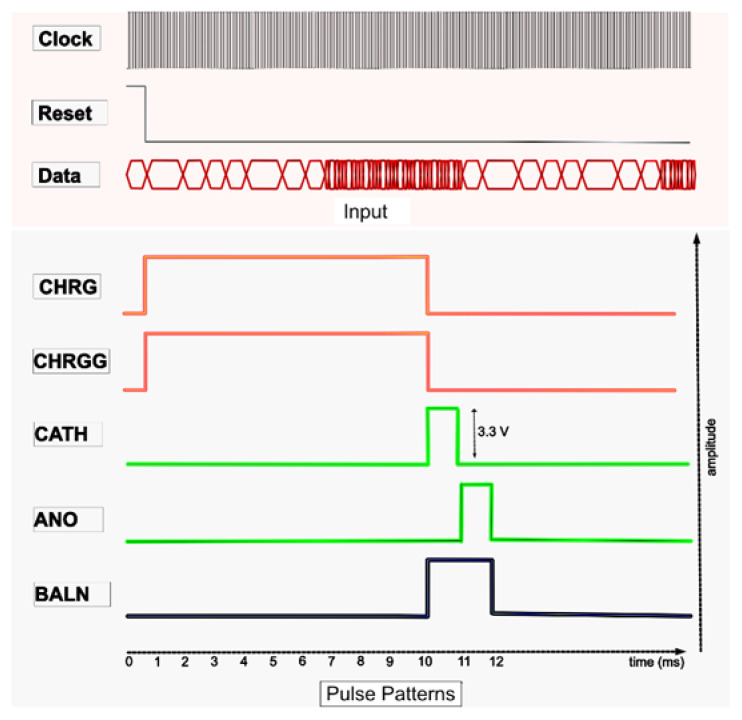
Pulse profiling.

Data from the transmitter were transferred into a wireless telemetry system that proceeded into the receiver FPGA. The receiver received data at a speed of 10 kbps, while the clock signal was received at a speed of 2 MHz. For the synchronization of data, the received clock output was internally divided into a clock of 10 kHz. The receiver generated various pulse profiles. Figure 5 shows the timing diagram of the pulse profiles.

The proposed digital block comprised four main blocks, which operated as follows:

- Generating current pulses in the pulse generator, including CHRG, CHRGG, CATH, ANO, and BALN;
- Timing of the CHRG and CHRGG pulses in the pulse-width controller block. CHRG and CRHGG were reset to the photodiode sensor. The amplitude of the light-dependent biphasic pulse was varied by controlling their width to digital pulses, which ranged between 1 to 10 ms;
- Controlling the timing and inter-pulse delay of CATH and ANO in the pulse sequence block. CATH and ANO generated cathodic and anodic currents of the biphasic pulse. In addition, different shapes of biphasic pulses, as shown in the results, could also be generated by varying the CATH and ANO pulses;
- The residual current was neutralized by turning on or off the BALN pulse;
- The stimulator array had four sets of channels, each of which comprised 16 pixels, as previously explained. Each channel set for stimulation was specified in the channel control block and merged with the width and delay control pulses in the channel-pulse merge block.

#### 2.2.5. Receiver Structure

**Figure 6:**
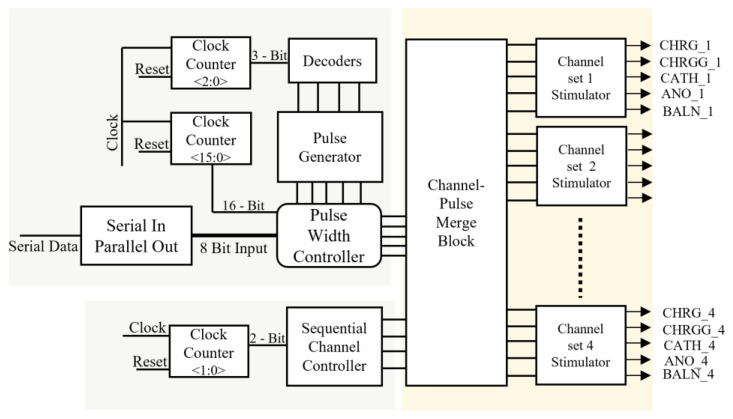
Detailed architecture of receiver.

Various blocks of the pulse profile generator are shown in Figure 6, which illustrate the operational process of the receiver, as described below.

##### Pulse Generator

This is a counter that takes the clock signal and resets it as inputs to generate a timing-controlled, two-bit output. Initially, the counter changed its state after the maximum time of a single-phase change, that is, 1 ms. A maximum interphase delay of 10 µs was used during the entire process. The output generated from the counter was wired into 3 × 8 decoders, which provided control logic for the number of pulses generated as cathodic-first pulses.

##### Channel Controller

This block provides sequential control logic for the stimulation of each channel set. A sequential channel controller received the input waveform from a two-bit clock counter and determined the specific channel set for each pulse. Four sets of channels were stimulated. Each set comprised 16 pixels.

##### Serial-to-Parallel Converter

This block received serial data from a wireless telemetry system. This block was designed in such a manner that it checks for the start of the data packet, followed by the header for verification of the data packet, and designates the address to each data packet; upon verification of a true data packet, it transfers seven-bit parallel data pulse-width controller, which varies the width of current pulses based on the value of data.

##### Pulse-Width Controller

The amplitude of the biphasic pulse can be clamped to a certain voltage level by controlling the stimulation timing of the CHRG. Sixteen CHRG pulses varied with a time difference of 62.5 µs, minimum width of 62.5 µs, and maximum default width of 1 ms, while widths of CATH and ANO varied from a minimum of 500 µs to a maximum of 1 ms, and the interval between both pulses varied from a minimum of 0 µs to a maximum of 1 ms, based on the increased or decreased value of seven-bit input obtained from data telemetry. The pulses varied depending on the seven-bit input pulse control received from the serial-to-parallel converter. A 16-bit clock divider was used to parse the pulse width, which shifted to its original time of 1 ms when reset to its initial value.

##### Merge Block

This block provided a pathway for each stimulus timing pulse for each designated channel set, based on the input signals received from the pulse width and channel controllers. The channel- and width-controlled pulses were the outputs of the analog circuit.

## 3. Results

### 3.1. Integrated Circuit (IC) Design

**Figure 7:**
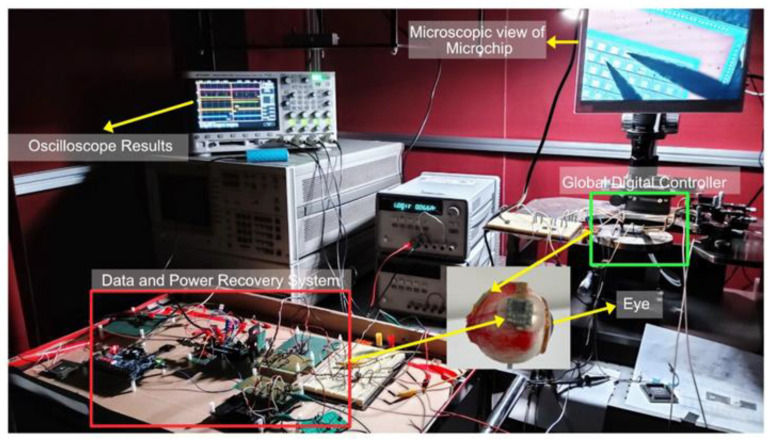
Prototype of the complete system and implanted chip on top of virtual eye.

**Figure 8:**
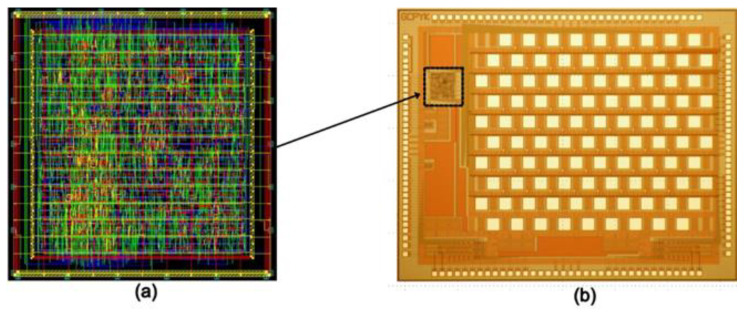
(**a**) Layout of digital controller embedded onto a chip (with an active area of 450 × 460 µm^2^); (**b**) microscopic view of 64-pixel fabricated microchip (with an active area of 4.3 × 3.2 mm^2^).

Design Vision e-2013 was used to synthesize the Verilog code and design layout after verifying the synthesized code. To manufacture an integrated circuit chip, a 0.35 μm SK Hynix CMOS standard process was used. A digital controller would be implanted inside the eye, which was fabricated on the same chip as a pixel stimulator, reducing the size and external connections. Figure 7 shows a photograph captured during the testing of the complete system. Figure 8 shows the layout generated and the fabricated chip.

### 3.2. COMSOL Simulations

This study aims to investigate the design of a stimulation pattern that prevents the loss of residual charge and minimizes cross-talk among the electrodes. Platinum electrodes were used as stimulators. Table 1 lists the basic parameters adopted for the electrode design. Standard parameters [25] for platinum electrode simulation were used based on its material properties.

**Figure 9:**
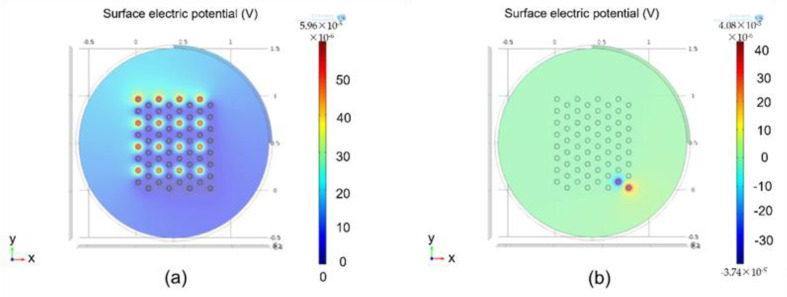
Simulation results calculated: (**a**) results calculated using COMSOL when a complete channel set was activated; (**b**) results calculated using COMSOL when two channels were activated simultaneously.

Figure 9 shows COMSOL simulation results. As shown in Figure 9a, 16 pixels were activated. Graphical description depicts that simulation of electrodes has a minimal effect on neighboring electrodes. COMSOL simulations prove the efficiency of our system in terms of cross-talk. Figure 9b shows the effect of stimulation of a single electrode when two channel sets were activated sequentially.

Compared with rectangular or conventional stimulation protocols, hexagonal stimulations prove to be an effective and efficient protocol, along with complete control of current by controlling pulse widths and shapes from the transmitter side.

### 3.3. ModelSim Simulations

**Figure 10:**
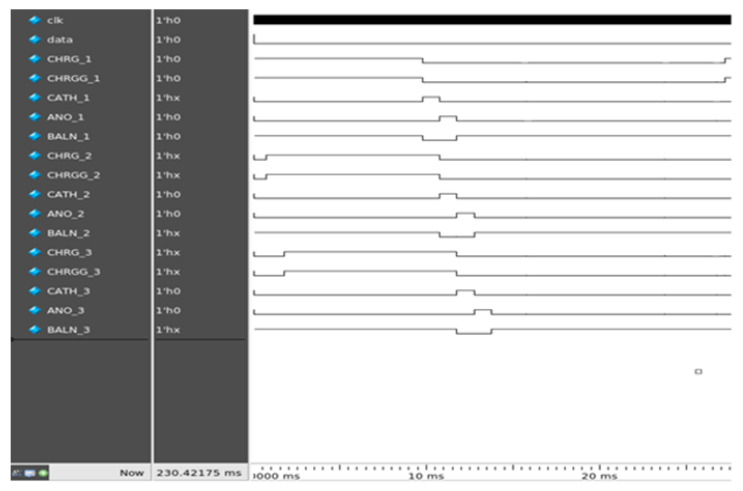
Simulation results calculated using ModelSim.

ModelSim v10.3e was used as the simulation tool for digital control. Figure 10 shows the simulation results of the digital controller on ModelSim for the two sets of channels. CHRG and CHRGG were simultaneously generated for 10 ms in this simulation. CATH and ANO, each for 1 ms, were consecutively generated to generate a cathodic-first pulse. A BALN pulse with a CHRG was produced.

### 3.4. Hardware FPGA and Microchip Implementation

**Figure 11:**
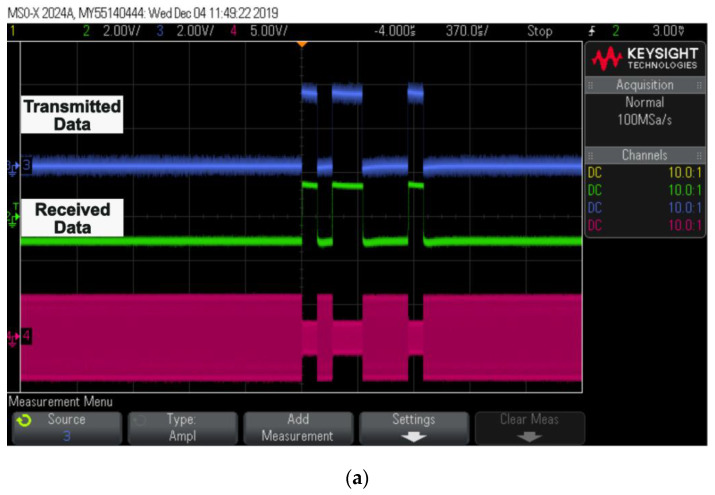

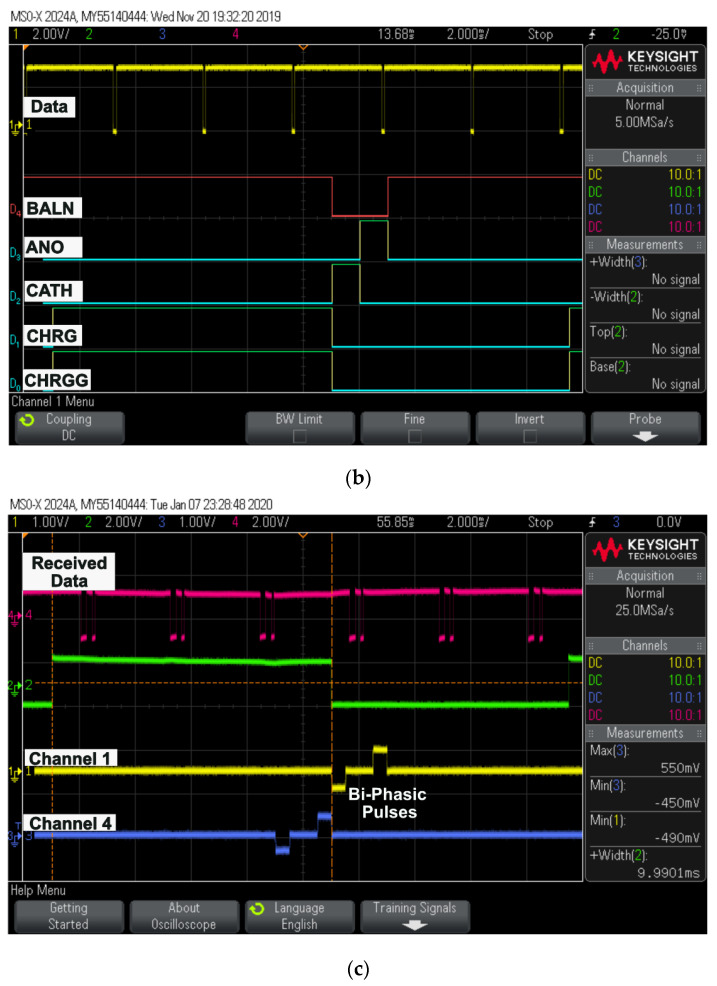
Oscilloscope results: (**a**) captured transmitted and received data; (**b**) digital pulse patterns generated using FPGA output; (**c**) generated biphasic pulses for two different channel sets with respect to received data.

Xilinx Vivado e-2018 was used to program an FPGA (Artix-7 BASYS 3). FPGA generated serial data and clock signals on the transmitter side, and these data and clock signals were used to generate pulse profiles on the receiver side. The results of hardware implementation are shown in Figure 11.

Transmitted and received data through a wireless telemetry system are displayed, along with ASK demodulated data, in Figure 11a. The data on both the transmitter and receiver sides were completely synchronized. Figure 11b shows the timing diagram of the current pulses using the FPGA. The width of the pulses can vary depending on the light source.

1. “D0” donates CHRG pulse;
2. “D1” donates CHRGG pulse;
3. “D2” donates CATH pulse;
4. “D3” donates ANO pulse;
5. “D4” donates BALN pulse.

Figure 11c shows biphasic pulses generated for two different channels sequentially on a digital controller designed and fabricated on 0.35 μm microchip technology with respect to the data.

## 4. Discussion

This study proposed the utilization of rectangular biphasic pulses using a fully controllable digital controller through a wireless transmission system for hexagonal stimulation. Previously designed architectures have used various stimulation protocols to simulate electrodes. However, they lacked efficiency in terms of current mismatch, power loss, and cross-talk effects, all of which play important roles in tissue damage and could ultimately lead to the failure of complete and expensive systems. Complex designs make these systems expensive and not commercially viable.

Implementation of rectangular biphasic pulses with light-dependent control over current helped our system counter the issues of current mismatch and power loss. Hexagonal stimulation using rectangular biphasic pulses with complete control over the amplitude and shape of rectangular biphasic pulses significantly leads to the reduction in cross-talk between the electrodes. This prevents unwanted stimulation of neighboring electrodes and would ultimately prove to be a solution for tissue damage during stimulation. Our system was verified through simulation and in vitro results using an FPGA and microchip for hardware verification. COMSOL simulations clearly depict the reduction in cross-talk among electrodes when hexagonal simulations were implemented. In addition, the digital controller provided complete control over the amplitude of the biphasic pulses using external data received wirelessly from the transmitter. The systems were designed for 64 pixels.

In the future, we plan to develop a more advanced and efficient system using advanced transmission protocols and a more enhanced digital controller. Upgrades would lead this system to be more precise, the vision would be improved by increasing the number of pixels, and risks related to tissue damage would be minimized to a large extent. We plan to design a system with 256 pixels and up to 2000 pixels.

## Figures and Tables

**Table 1:** Table lists basic parameters used for electrodes COMSOL simulations.

S.No	Property	Name	Value	Unit
1	Electrical conductivity	Sigma	8.9 × 10^6^ (S/m)	S/m
2	Coefficient of thermal expansion	alpha	8.80 × 10^−6^ (1/K)	1/K
3	Heat capacity at constant pressure	Cp	133 (J/(kg·K))	J/(kg·K)
4	Density	rho	21,450 (kg/m^3^)	kg/m^3^
5	Thermal conductivity	k	71.6 (W/(m·K))	m·K

## Data Availability

The data presented in this study are included in this paper.
